# Surgical Antibiotic Prophylaxis in Small Animal Surgery: A Retrospective Outcome-Based Study from the Veterinary Teaching Hospital of Naples

**DOI:** 10.3390/ani15111600

**Published:** 2025-05-30

**Authors:** Stefano Cavalli, Chiara Caterino, Francesca Paola Nocera, Giovanni Della Valle, Rossana Schena, Federica Aragosa, Francesca Pizzano, Luisa De Martino, Gerardo Fatone

**Affiliations:** Department of Veterinary Medicine and Animal Production, University of Naples Federico II, Via F. Delpino 1, 80137 Naples, Italy; stefano.cavalli@unina.it (S.C.); chiara.caterino@unina.it (C.C.); giovanni.dellavalle@unina.it (G.D.V.); rossana.schena@unina.it (R.S.); federica.aragosa@unina.it (F.A.); francesca.pizzano@unina.it (F.P.); luisa.demartino@unina.it (L.D.M.); fatone@unina.it (G.F.)

**Keywords:** antibiotics, small animals, surgery rooms, surgical site infections

## Abstract

Hospital-acquired infections in operating rooms are contributing factors to the increasing presence of pathogens, with surgical site infections being the most frequent among them. Surgical antimicrobial prophylaxis is administered to minimize the risk of developing surgical site infection. Surgeons are responsible for weighing the benefit of surgical site infection-risk reduction for the individual patient afforded by prophylactic antimicrobial administration against the broader societal risk from such use. The aim of this study was to analyze the data on surgical antimicrobial prophylaxis related to patient status, surgery duration, wound classification and the occurrence of surgical site infection in two operating rooms of the Veterinary Teaching Hospital of Naples from January 2023 to January 2024. Two hundred seventy-seven surgical procedures met the inclusion criteria. For soft tissue clean-contaminated procedures and clean orthopedic surgeries not involving implants, no statistically significant difference was observed between the various antibiotic protocols used and surgical site infection; this finding supports the possibility of avoiding antibiotic use for these procedures. In contrast to previous reports, this study identified a statistically significant association between ASA status and surgical site infection; no correlation was found between surgical duration and the occurrence of infection. Further research on antibiotic use and risk factors associated with surgical site infection will be crucial to strengthen clinical guidelines.

## Simple Summary

Antimicrobial resistance is one of the most significant global health threats. The use of antibiotics contributes to the development of resistance, making it crucial to administer them only when necessary. Antibiotic guidelines require scientific studies to determine where and when antibiotics should be used. The aim of this retrospective study was to assess how antibiotic prophylaxis influenced the incidence of surgical site infections and to identify the main risk factors in 277 patients who underwent open surgical procedures in the Veterinary Teaching Hospital of Naples. For the first time in veterinary medicine, this study found a strong statistical correlation between a patient’s status, as classified by the American Society of Anesthesiologists, and surgical site infection. Additionally, consistent with previous studies, it confirmed that prophylactic antibiotic administration is unnecessary for certain types of surgeries. Future prospective and retrospective studies that adhere to classification criteria proposed by guidelines will help strengthen the scientific evidence regarding the proper use of antibiotics in veterinary practice.

## 1. Introduction

Since their discovery, antibiotics have played an essential role in the treatment of numerous infectious diseases, helping to improve the health of people and animals significantly [1]. Today, this improvement risks being undermined by the growing spread of pathogens resistant to them. All antimicrobial use contributes to antimicrobial resistance (AMR), but in recent years, the phenomenon of resistance has been amplified and accelerated by the excessive and improper use of antibiotics, in both human and veterinary medicine. The selective pressure on the microbial population can produce deleterious effects on the health of humans and animals due to the loss of effectiveness of therapies, with consequent risk of greater severity and spread of diseases [2,3].

The issue of healthcare-associated infections and the emergence of zoonotic and multidrug-resistant pathogens in companion animal medicine, particularly for dogs and cats, represent significant challenges that have been recognized for decades. However, the implementation of effective infection control programs in veterinary clinics has seen slow progress, which may be due to several factors, such as lack of standardized guidelines, resource limitations and emerging pathogen complexity [4].

Hospital-acquired infections or contamination in operating rooms (ORs) contribute to the increasing presence of pathogens in human and veterinary clinical samples. Surgical site infections (SSIs) are the most frequent hospital-acquired infections, accounting for 20% of all human hospital-acquired infections [5]. Although comparable reporting of nosocomial infections is lacking in veterinary medicine, SSIs have been reported as a complication in 0.8% to 18.1% of small animal surgeries, with significant variations depending on the type of procedure [6,7]. SSIs cannot be eliminated, but preventive strategies represent the most effective means of reducing their impact. These strategies include adherence to aseptic protocols during surgery, the judicious use of surgical antimicrobial prophylaxis (SAP), identification of at-risk patient populations, and correct surgical wound management in the postoperative period [8]. When SSI occurs, accurate and timely identification of infection, appropriate assessment of the extent of infection, culture-based antibiotic therapy, appropriate wound management, and attention to infection control protocols are essential to ensure the best outcome. Finally, surveillance protocols are critical to identify systematic breaks in surgical asepsis, inadequate perioperative care protocols, and antimicrobial resistance patterns [9].

In veterinary medicine, SAP is the administration of antimicrobials to animals undergoing a surgical procedure to prevent the development of an SSI and does not include preoperative decolonization or treatment of established infections [8,10]; it should be used judiciously to balance the prevention of infections with the risks of overuse, including AMR, dysbiosis and adverse reactions. The administration of antibiotics in surgical procedures for pet animals follows principles similar to human medicine but with considerations specific to veterinary care. As in human medicine, antibiotics should ideally be administered 30–60 min before the first incision to ensure adequate tissue concentrations by the time the surgery begins. This helps prevent infection by reducing bacterial load at the surgical site [8,11]. Unfortunately, compared to human medicine, there is no clear evidence in the specific guidelines for the use of antimicrobials in veterinary medicine. A key gap in veterinary literature is a lack of randomized control trials on SAP in common veterinary surgical procedures [10].

Another significant gap in the literature, highlighted by Sorensen et al. [10], is the lack of defined standards for comparing antibiotic data. Some authors consider therapy administered within 24 h from skin closure as perioperative SAP, while others classify it as postoperative therapy [10].

This discrepancy makes it difficult to analyze and compare the available data, underscoring the need for further outcome-based studies. Among the challenges in establishing clear evidence on antibiotic prophylaxis, the considerable variability of factors predisposing to surgical site infection should also be acknowledged. These include host susceptibility, surgical duration, type of surgery, degree of wound contamination, concurrent diseases, and other factors [8].

To date, for clean and clean-contaminated procedures that do not involve implants, some guidelines recommend either avoiding surgical antimicrobial prophylaxis (SAP) altogether or limiting its use to perioperative prophylaxis (peri-SAP) without continuation beyond surgery. Postoperative prophylaxis (post-SAP), defined as the continuation of perioperative prophylaxis beyond 24 h after skin closure, is generally reserved for orthopedic surgeries involving implants, central nervous system surgeries, and surgeries where the consequences of septic damage could be severe [8,10,12].

Despite guidelines for SAP in veterinary surgery being recently published [8,10], there is still a need for outcome-based studies using reproducible data, following the latest criteria for SAP, SSIs, surgical wound classification, surgical time, and ASA patients’ status. The lack of supportive clinical data represents a significant limitation of most recommendations in small-animal guidelines [13]. Only when the same criteria are applied across studies will the data be comparable and the results reliable.

The objective of this study was to analyze the data on SAP peri- and postoperative, correlated to patient status, time of surgery, wound classification and the occurrence of SSIs for surgeries performed at two ORs of the Veterinary Teaching Hospital (VTH) of Naples from January 2023 to January 2024.

## 2. Materials and Methods

The study population included client-owned dogs and cats referred daily to the hospital to undergo surgical procedures. The medical records of patients who underwent surgical procedures at the VTH of Naples from January 2023 to January 2024 were retrospectively reviewed.

The study included patients who underwent open surgical procedures under general anesthesia and had an adequate follow-up, defined as at least six months for soft tissue surgery and at least one year for orthopedic and neurosurgical procedures. Only cases with complete medical records containing detailed information on antimicrobial treatments, type of surgery, surgical site infections, and the American Society of Anesthesiologists (ASA) classification were included. Additionally, cases were included only if their data could be classified according to the Centres for Disease Control and Prevention (CDC) classification. Approval by an ethical committee was not required for this study, as all procedures were consistent with routine veterinary practice. Informed consent was obtained from all owners prior to any diagnostic or therapeutic intervention necessary for the management of the condition for which the animals were referred.

### 2.1. Data Collection

For each animal, information regarding the ORs used was recorded. Operating Room 1 (OR1) in our hospital is designated for soft tissue surgery, while Operating Room 2 (OR2) is designated for orthopedic and neurosurgical procedures.

Data regarding surgery type and duration (greater or less than 60 min), level of wound contamination according to the CDC classification [14], host susceptibility according to ASA classification, and SSIs, according to the CDC classification, were collected.

Follow-up evaluations included periodic evaluations by the surgeon, follow-up assessments by the attending internist after surgery, and telephone consultations with the owner.

### 2.2. Veterinary Antibiotic Prescriptions

A retrospective cross-sectional design was conducted for this study. All data concerning the veterinary antibiotic prescriptions of the surgery unit for each animal related to the two phases of antibiotic treatment (peri-SAP and post-SAP) were collected by a software called “Myclinical” (software version 18.3.1, MG Soft Srls—Salerno, Italy), a cloud-based electronic health record system.

### 2.3. Data Management and Statistical Analysis

The collected data were recorded in a data-capturing format and then input into a Microsoft 365 Excel™ spreadsheet for further analysis.

The association between categorical variables and the occurrence of SSI was assessed using Fischer’s Exact Test. *p* values < 0.05 were considered statistically significant at a 95% confidence interval. Analyses were performed using the Statistical Program IBM Corp. Released in 2019. IBM SPSS Statistics for Windows, Version 26.0. Armonk, NY, USA: IBM Corp.

To facilitate statistical analysis and ensure adequate group sizes, the variables were dichotomized as follows: ASA status grouped into low ASA (I and II) and high ASA (III to V), wound classification grouped into clean/clean-contaminated versus contaminated/dirty, duration of surgery >60 min and ≤60 min, and antibiotic protocol divided into peri-SAP + post-SAP and others protocols (peri-SAP only, post-SAP only and no antibiotics).

A Chi-square test was performed to assess the correlation between each ASA status grade and the incidence of SSI.

## 3. Results

Data were collected from 277 surgical procedures that met inclusion criteria: 233 were performed on dogs, 39 on cats, 3 on rabbits and 2 on guinea pigs. Table 1 and Table 2 show the surgical procedures performed in OR1 (soft tissue) and OR2 (orthopedic and neurosurgery), respectively.

Patients’ ages ranged from 1 month to 18 years, comprising 113 females and 164 males. A total of 56 patients were neutered, while 221 were intact.

### 3.1. Surgical Procedures

In OR1, 178 soft tissue surgeries were performed, and the procedures mainly included genitourinary, respiratory, and gastrointestinal procedures. Minimally invasive soft tissue surgeries were also performed in OR1 but were excluded from the study (Table 1).

The most performed surgical procedures in the OR1 involved the genitourinary system (50 surgeries, 28%), with urinary system procedures being the most frequent, such as cystotomies, urethrotomies, and urethrostomies; the second most performed surgical procedures involved the gastrointestinal system (41 surgeries, 23%), including enterotomies, enterectomies, and gastrectomies (Table 1).

In OR2, 99 orthopedic and neurosurgical surgeries were performed (Table 2); these surgeries were performed in an operating room classified as ISO 6 category (UNI EN ISO 14644-1:2016 [15]), which defines standards for clean rooms and controlled environments, particularly in terms of the concentration of airborne particles. Minimally invasive surgeries, such as arthroscopy, were also performed in OR2 but were excluded from the study.

The most commonly performed surgical procedures in OR2 are implant-related orthopedic surgeries (81 procedures, 82%), including fracture repairs and tibial tuberosity advancement (TTA), tibial plateau levelling osteotomies (TPLO) and tibial tuberosity transposition (TTT). Neurosurgical procedures involved laminectomies and one craniotomy (Table 2).

### 3.2. Surgical Time

The medical records were analyzed to record the surgical time of each procedure. Table 3 shows the duration of surgeries in OR1 (soft tissue) and OR2 (neurosurgical and orthopedic). In OR1, 119 procedures (67%) lasted more than 60 min, while 59 (33%) lasted 60 min or less. In OR2, 96 procedures (97%) lasted more than 60 min, while the remaining 3 (3%) lasted 60 min or less.

### 3.3. Wound Classification

According to CDC classification, data about wound classification were collected from medical records and reported in Table 4. In OR1, 79 procedures were classified as clean-contaminated, 42 as contaminated, 38 as clean and 19 as dirty. In OR2, 96 procedures were classified as clean, 82% of which involved implant-related orthopedic surgery, 1 was classified as contaminated, and 2 open fractures as dirty.

### 3.4. ASA Status and Patients’ Comorbidity

In OR1, 10 patients were classified as ASA I, 120 as ASA II, 36 as ASA III, 9 as ASA IV, and 3 as ASA V. In OR2, 10 patients were classified as ASA I, 83 as ASA II, 6 as ASA III, and no patients were classified as ASA IV or ASA V.

None of the patients who developed SSI had comorbidities or bacterial conditions requiring antibiotic therapy. A dog who developed SSI had splenic hemangiosarcoma. None of the patients included in this study were on corticosteroids.

### 3.5. Antibiotic Treatment

As shown in Table 5, peri-SAP and post-SAP were administered to 101 patients in OR1 and 94 in OR2; only peri-SAP was given to 45 and 2 patients in OR1 and OR2, respectively.

Post-SAP without peri-SAP was administered to 17 patients in OR1, while no patient received post-SAP without peri-SAP in OR2. No antibiotic was administered to 15 and 3 patients in OR1 and OR2.

In all patients, post-SAP was administered to prevent SSI, serving as a continuation of peri-SAP in combination with antibiotic treatment used as comorbidity treatment (e.g., a patient with a foreign body and leptospirosis).

The most used antibiotics for peri-SAP in both OR1 and OR2 were ampicillin (38%), amoxicillin (29%), and cefazolin (16%). For post-SAP, the most frequently administered antibiotic was amoxicillin-clavulanic acid (81%), followed by cefazolin (7%).

In 21 patients, the timing of antibiotic prophylaxis administration did not comply with current guidelines, with prophylaxis initiated between 4 and 1 days before skin incision or the first dose delayed after incision by 10 to 60 min. Post-SAP duration ranged from 3 to 12 days, depending on surgical procedures.

According to the European Medicines Agency (EMA) categorization, no antibiotics belonging to class A (avoid) were administered. Antibiotics belonging to class B were administered in 14.6% of the treated patients, with enrofloxacin being the most used antibiotic for this category. Antibiotics belonging to class C and D (caution and prudence, respectively) were used in 85.4% of treated patients, with amoxicillin-clavulanate as the most used. Where it was not possible to perform an antibiogram, molecules of class D and C (prudence and caution, respectively) were chosen. An antibiogram was always performed before administration for class B molecules (restrict) or a dual agent treatment.

The results showed no apparent toxicities associated with the administered doses of antibiotics. A dog, a male Cocker Spaniel, aged 10, developed an adverse reaction immediately after a single administration of marbofloxacin intravenously at 2 mg/kg.

### 3.6. Surgical Site Infections

Ten patients (3.6%) developed SSIs, classified as superficial incisional [3], deep incisional [3], and organ/space infections [4] as reported in Table 6 Specifically, 4 out of 99 patients (4.0%) in OR2 and 6 out of 178 (3.4%) in OR1 developed SSIs.

For each patient who developed an SSI, the type of infection, age, ASA status, wound classification, type and duration of surgery, and antibiotic treatment (peri-SAP and post-SAP) were analyzed and reported in Table 6. As shown in Table 6, patients who developed SSIs were identified with sequential numbers from 1 to 10.

All patients with SSIs were dogs. Among clean procedures, 2 out of 134 cases (1.4%) resulted in SSIs, while among contaminated procedures, 5 out of 43 cases (11.6%) developed SSIs. In dirty procedures, SSIs occurred in 3 out of 21 cases (14.3%), all classified as organ/space infections. In case 10, a dog with a linear foreign body and intestinal perforations, the patient died four days after surgery.

ASA status was classified as IV in 4 patients, III in 3 patients, and II in 3 patients who developed SSI.

Only patient 4 had comorbidities, as the cause of hemoabdomen was a splenic hemangiosarcoma with lymph node metastases.

Among clean procedures performed in OR1, no patients developed SSI, regardless of whether they received peri-SAP and post-SAP, peri-SAP alone, post-SAP alone, or no antibiotic therapy. In OR2, two patients underwent clean procedures and developed SSI.

Among clean-contaminated procedures, no patients developed SSI in OR1 and OR2, regardless of whether they received peri-SAP and post-SAP, peri-SAP alone, post-SAP alone, or no antibiotic therapy.

All SSIs following contaminated procedures occurred in patients who received both peri- and post-SAP. Similarly, all dirty procedures were managed with peri- and post-SAP.

The mean follow-up period was 8 months for soft tissue procedures (ranging from 6 to 11 months) and 13 months for orthopedic and neurosurgical procedures (ranging from 12 to 16 months).

### 3.7. Statistical Analysis

In OR1, statistical analysis revealed a significant association between wound classification and the occurrence of SSI, comparing contaminated and dirty wounds to clean or clean contaminated, with a *p*-value of 0.0014. A statistically significant association was found between ASA status and SSI, with patients classified as III to V showing a higher infection rate than ASA I and II (*p* = 0.0059). No statistically significant association was found between the duration of surgery and SSI (*p* = 1.0000). Comparison between peri-SAP and post-SAP and other antibiotic protocols showed a statistically significant association with SSI occurrence (*p* = 0.0371).

In OR2, for orthopedic e neurological surgery, Fisher’s Exact Test revealed a statistically significant association between wound classification (contaminated/dirty vs. clean/clean-contaminated) and SSI occurrence (*p* = 0.0037). A statistically significant association was also found between ASA status (high ASA III to V and low ASA I and II) and SSI (*p* = 0.0190). No statistically significant association was found between the duration of surgery and SSI (*p* = 0.1180). No statistically significant difference was found between antibiotic protocols and SSI (*p* = 1.0). For ASA status, evaluating each ASA grade individually, the Chi-square statistic was 29.94 (*p* < 0.0001).

## 4. Discussion

Based on the aim of this study, the use of surgical antimicrobial prophylaxis (SAP) in operating rooms (ORs) was analyzed for both soft tissue surgeries and orthopedic and neurosurgical procedures. The analysis accounted for several variables that, in addition to SAP, may influence the occurrence of surgical site infections (SSIs). The observed SSI incidence was consistent with rates reported in previous studies [7,16,17,18,19].

Among the variables examined, wound classification showed a statistically significant association with SSI occurrence for soft tissue (*p* = 0.0014) and orthopedic and neurosurgery (*p* = 0.0037), confirming the hypothesis that wounds classified as contaminated or dirty increase the likelihood of SSI in both soft tissue and orthopedic/neurosurgical procedures.

Another important finding is the correlation between wound classification and the severity of SSI. All patients who developed SSI with a wound classified as dirty developed an organ/space SSI, the most severe form of SSI.

Conversely, surgical duration, which has been previously identified as a factor influencing SSI risk [8,20,21,22,23], was not statistically correlated with SSI occurrence in this study, neither for soft tissue surgeries (*p* = 1.0000) or orthopedic and neurosurgical procedures (*p* = 0.1180).

Previous studies showed that ASA classification was not a risk factor for developing SSI [17,20]. However, in human medicine, ASA classification is considered a proven risk factor for developing SSI by the Consensus Supervision of Surgical Wound Infection [24].

The results of this study did not align with recent veterinary studies, as a statistically significant association was found between ASA status and SSI in soft tissue (*p* = 0.0059) and orthopedic/neurosurgical surgeries (*p* = 0.0190). A Chi-square test was performed to assess each ASA grade’s direct correlation, considering each grade (I to V) individually. The results confirmed the statistical significance between ASA status and SSI (*p* < 0.0001).

To the authors’ knowledge, the correlation between ASA classification and SSI has, until now, been based solely on human medicine antibiotic guidelines. The findings of this study offer novel evidence in the veterinary field supporting the association between ASA classification and the risk of SSI, supporting its inclusion in veterinary risk assessment models for SSI.

All patients included in the study have complete SAP data. Among soft tissue, for clean and clean-contaminated surgery, peri-SAP and post-SAP were administered to 46 patients, peri-SAP and without post-SAP to 43 patients, post-SAP without peri-SAP to 14 patients and no antibiotic prophylaxis to 14 patients. The data suggested that, despite the good variability in procedure type, ASA status, and time of surgery, antibiotic prophylaxis did not reduce the risk of SSI in soft tissue clean and clean-contaminated surgeries. None of the patients developed SSI, regardless of the antibiotic protocol used. This finding is in concordance with the recent guidelines [8,21], but it is in contrast with the findings of Eugster et al. [7], which showed that preoperative use of antimicrobial prophylaxis, even in clean procedures, acts as a protective factor against the occurrence of SSI.

For procedures classified as contaminated, nearly all patients received peri-SAP and post-SAP per current guidelines. Only three patients received a dual-agent therapy (amoxicillin + enrofloxacin) post-SAP, one of whom developed an SSI. Due to the limited sample size, further studies are needed to determine whether dual-agent therapy may influence the occurrence of surgical site infections, considering the adverse effects, including dysbiosis and AMR.

In OR1, according to the wound classification proposed by the CDC, 12 out of 38 (31.6%) clean procedures were compliant with recent guidelines, with no antibiotics administered either as pre- or post-operative prophylaxis. Among the clean-contaminated procedures, 31 out of 79 (39.2%) followed recent guidelines, as peri-SAP were administered without continuation into the postoperative period. Regarding contaminated procedures, 36 out of 42 (85.7%) aligned with the guidelines. A single case of a contaminated wound caused by a chronic wound of unknown origin was treated with L-PRF according to the protocol proposed by Caterino et al. [25] and Aragosa et al. [26] without administration of perioperative or postoperative SAP.

Finally, all 19 dirty procedures (100%) adhered to recent perioperative and postoperative antibiotic use guidelines.

In OR2, all procedures were in concordance with recent guidelines. Peri-SAP and post-SAP were administered for all orthopedic procedures involving implants and neurosurgical procedures on the central nervous system. No antibiotic prophylaxis or treatment was used for orthopedic surgeries that did not involve implants.

For procedures classified as dirty, 1 out of 19 (5.2%) developed SSI in OR1, while 2 out of 2 (100%) developed SSI in OR2. All the patients received peri-SAP and post-SAP, which concurs with current guidelines on antimicrobial use in small animal practice.

Although the sample size is very limited, the 100% incidence of SSI in dirty wounds (osteomyelitis) in OR2 highlighted the need to consider multiple factors that may influence infection risk carefully. In particular, the choice of implants (internal plate versus external fixation), the SAP, the hospitalization and other factors should be critically evaluated. The findings of this study for OR2 on SAP in orthopedic and neurosurgical procedures were fully consistent with DSAVA and ENOVAT guidelines [8,10], which recommend antibiotic SAP for implant-related procedures and no antibiotic treatment for clean, non-implant-related orthopedic procedures. In this study, three non-implant-related orthopedic procedures were performed, and none of the patients developed SSI despite the absence of SAP. Despite the limited sample size, the lack of SSIs observed suggests that antibiotic prophylaxis might not provide significant advantage in these cases.

For implant-related procedures, little evidence supports the use of perioperative and postoperative SAP. Budsberg et al. [12] evaluated the efficacy of postoperative antibiotic use following TPLO in dogs and found little supporting evidence. In accordance with current guidelines, peri-SAP and post-SAP were administered to all patients undergoing implant orthopedic and neurological procedures, and the observed SSI rate was comparable to previous studies.

All patients who developed a surgical site infection received SAP with the first administration correctly timed 30–60 min before the skin incision, in accordance with current guidelines. Therefore, this study does not allow for an evaluation of whether improper timing of SAP initiation could influence the occurrence of SSI.

The three antibiotics frequently used in SAP administered in this study, with dosing based on weight, were amoxicillin-clavulanate, ampicillin, and cefazolin. Amoxicillin-clavulanate was the most commonly administered antibiotic as postoperative SAP in both ORs, whereas ampicillin and cephazolin were the most used as perioperative SAP; however, a greater variability in the choice of antibiotics has been reported for the antibiotics administrated after surgery. In any case, broad-spectrum antibiotics, including penicillin with lactamase inhibitors, as well as first-generation cephalosporins, were already reported as the most frequently prescribed compounds in a previous study performed at Naples VTH, related to the traceability system of veterinary antimicrobial prescriptions [27].

Many clinical studies have already assessed the efficacy of amoxicillin-clavulanate as an antibiotic in surgery for its broad spectrum of actions against Gram-positive and Gram-negative bacteria and anaerobic pathogens that have a relevant role in postoperative infections [28,29,30]. The use of cefazolin was supported by the study conducted by Pelligand et al. [31], which highlighted that cefazolin is the only broad-spectrum antibiotic recommended for SAP with activity against *E. coli*.

In this study, class C and D antibiotics, according to EMA classification, were used in 90% of cases. Consequently, it was impossible to determine whether broad-spectrum antibiotics from these classes are adequate for effective SAP, potentially eliminating the need for agents from other classes not recommended by the EMA.

This investigation has several limitations. Firstly, its retrospective, single-center design confines the findings to a specific geographic location, potentially limiting generalizability to broader populations. The retrospective nature also restricted the inclusion of all surgical procedures performed at the VTH, resulting in a reduced sample size, particularly within certain procedural categories. Additionally, the study’s design constrained access to intraoperative cardiorespiratory data during anesthesia, precluding their incorporation into the statistical analysis. Furthermore, the low incidence of SSIs relative to the sample size impeded the execution of a robust multivariate analysis, which could have more effectively elucidated the interplay of factors contributing to SSIs.

## 5. Conclusions

In conclusion, retrospective outcome-based studies are crucial in guiding antimicrobial stewardship programs, particularly when standardized parameters such as CDC classifications are applied. This study supports the introduction of ASA status as a risk factor based on human studies and by providing evidence in veterinary medicine. However, further research with larger and more geographically diverse samples is needed to strengthen the reliability of these findings. Additionally, prospective studies will be essential to assess the impact of different SAP protocols better and refine evidence-based recommendations for surgical antibiotic use.

## Figures and Tables

**Table 1:** Surgical procedures performed in OR1 (soft tissue).

Surgical Procedures	Number of Surgeries	Percentage of Surgeries
**Genitourinary**	50	28%
**Gastrointestinal**	41	23%
**Dermatological**	17	9%
**Respiratory**	16	9%
**Dental**	13	7%
**Ophthalmological**	13	7%
**Ear**	13	7%
**Spleen**	10	6%
**Endocrine**	3	2%
**Biliary**	2	1%

**Table 2:** Surgical procedures performed in OR2 (orthopedic and neurosurgery).

Surgical Procedures	Number of Surgeries	Percentage of Surgeries
**Orthopedic surgery involving implants**	81	82%
**Neurosurgery**	13	13%
**Orthopedic surgery not involving implants**	5	5%

**Table 3:** Surgical duration of procedures performed in OR1 and OR2.

Surgical Time	OR1	OR2
Time > 60 min	119	96
Time ≤ 60 min	59	3

**Table 4:** Wound classification for each procedure performed in OR1 and OR2.

Wound Classification	OR1	OR2
**Clean**	38	96
**Clean-contaminated**	79	0
**Contaminated**	42	1
**Dirty**	19	2

**Table 5:** The antibiotic treatment administered in patients in OR1 and OR2.

Antibiotic Treatment	OR1				OR2			
	**Clean**	**Clean-** **Contaminated**	**Contaminated**	**Dirty**	**Clean**	**Clean-** **Contaminated**	**Contaminated**	**Dirty**
**peri-SAP and post-SAP**	9	37	36	19	92	0	0	2
**Peri-SAP and no post-SAP**	12	31	2	0	1	0	1	0
**No peri-SAP and post-SAP**	5	9	3	0	0	0	0	0
**No antibiotic treatment**	12	2	1	0	3	0	0	0

**Table 6:** Data recorded for each patient who developed SSI. Type of SSI, age, ASA status, wound classification, type of surgery, time of surgery, and antibiotic treatment (peri-SAP and post-SAP). Type of surgical site infections are divided into the superficial incision (S), a deep incision (D) or organ/space, (OS), following the CDC classification.

Patient ID	Type of Surgery	Age	ASA Status	Wound Classification	Type of SSI	Time of Surgery	Peri-SAP	Post-SAP
**1 (Dog)**	Gastric dilatation and volvulus	5 years	IV	Contaminated	D	>60 min	Ampicillin	Amoxicillin − clavulanic acid
**2 (Dog)**	Open fracture	8 months	III	Dirty	OS	>60 min	Amoxicillin	Amoxicillin + Enrofloxacin
**3 (Dog)**	Open fracture	8 months	III	Dirty	OS	<60 min	Amoxicillin + Enrofloxacin	Amoxicillin + Enrofloxacin
**4 (Dog)**	Hemoabdomen	11 years	IV	Contaminated	OS	>60 min	Ampicillin	Amoxicillin + Enrofloxacin
**5 (Dog)**	Exploratory laparotomy	11 years	IV	Contaminated	OS	<60 min	Ceftriaxone	Ceftriaxone
**6 (Dog)**	Enterectomy	9 years	III	Contaminated	S	>60 min	Cefazolin	Cefazolin
**7 (Dog)**	Dog bite wound >10 cm	8 years	II	Contaminated	D	<60 min	Amoxicillin	Amoxicillin
**8 (Dog)**	Hemilaminectomy	10 years	II	Clean	D	>60 min	Clindamycin	Clindamycin
**9 (Dog)**	Hemilaminectomy	9 years	II	Clean	S	>60 min	Ampicillin	Ampicillin
**10 (Dog)**	Linear foreign bodywith intestinal perforation	3 years	IV	Dirty	OS	>60 min	Cefazolin	Cefazolin+ Amoxicillin

## Data Availability

The original contributions presented in this study are included in the article.

## Abbreviations

The following abbreviations are used in this manuscript:

AMR	antimicrobial resistance
ORs	operating rooms
SSIs	surgical site infections
SAP	surgical antimicrobial prophylaxis
Peri-SAP	perioperative prophylaxis
Post-SAP	postoperative prophylaxis
VTH	Veterinary Teaching Hospital
CDC	Centers for Disease Control and Prevention
ASA	American Society of Anesthesiologists
OR1	operating room 1
OR2	operating room 2
EMA	European Medicines Agency
